# Comparing potential early caries assessment methods for teledentistry

**DOI:** 10.1186/1472-6831-13-16

**Published:** 2013-03-28

**Authors:** Zachary Van Hilsen, Robert S Jones

**Affiliations:** 1 School of Dentistry, University of Minnesota, Minneapolis, MN 55455, USA; 2 Division of Pediatric Dentistry, School of Dentistry, University of Minnesota, 6-150 Moos Health Science Tower, 515 Delaware Street S.E., Minneapolis, MN 55455, USA

**Keywords:** Telehealth, Caries, Imaging, Optical Coherence Tomography, Detection system, Light-Emitting Diode

## Abstract

**Background:**

Optical caries detection has the potential to be incorporated in telehealth medicine for preventive dental screening. The objective of this study was to evaluate and compare visible and near infrared detection methods for identifying early non-cavitated *ex vivo* occlusal demineralization.

**Methods:**

Six blinded examiners were used to compare the accuracy of the following three examinations in detecting occlusal demineralization: Midwest Caries ID™ (MID), visual photographic examination (CAM) and Cross Polarization Optical Coherence Tomography (CP-OCT). For each diagnostic method, two examiners assessed the extracted tooth samples 1–2 weeks apart. Teeth were then sectioned and lesion depth was confirmed (n = 42) by a blinded histological examination using a glycol based caries indicator dye. The sensitivity (Sen), specificity (Sp), Intraclass Correlation Coefficient (ICC), and Area under the Receiver Operator Curve (AUC) were calculated.

**Results:**

For detecting any demineralization versus sound pit and fissure enamel, the mean Sen/Sp found was 46.9/85.0 for MID, 80.5/52.5 for CAM, and 83.4/45.0 for CP-OCT. For detecting non-cavitated demineralization that progressed into the dentin, the mean Sen/Sp found was 17.3/88.0 for MID, 48.0/57.8 for CAM, and 44.2/72.7 for CP-OCT. AUC values were statistically significant (P < 0.05) in three out of four examiner assessments when MID and CP-OCT were used to detect any demineralization. AUC values were significant for a single CAM examination. When assessing deeper non-cavitated lesions, none of the assessment methods were able to yield AUC values that were significantly different than a random ‘coin flip’ test. When examining reliability, MID demonstrated the highest ICC score (0.83) and CP-OCT had the lowest (0.49).

**Conclusion:**

Although MID and CP-OCT were useful in detecting the presence of demineralization, examiners were not able to utilize these devices to adequately assess the depth of the demineralization. This study found that MID and CP-OCT did not have markedly superior diagnostic values from simple CAM assessment for use in teledentistry.

## Background

Preventive dental care has the potential to be delivered by adjunct dental personnel in remote or critical access areas. Children with a high-risk for dental caries may be effectively screened for early presence of non-cavitated lesions utilizing telehealth technologies [1,2]. Children can be screened with an intraoral camera in teledentistry where images are collected by trained adjunct dental personnel. Images are then interpreted and assessed remotely by a dentist. Teledentistry may also include caries risk assessment questionnaires and objective caries detection measurements that are forwarded to a dentist for a comprehensive evaluation. Since caries on fissured surfaces account for over 80% of all caries on young permanent teeth [3], dental radiography may not effectively identify early non-cavitated lesions on critical tooth surfaces. Newer optical methods, such as the Midwest Caries ID™ (MID) and CP-OCT, have the potential to detect early non-cavitated occlusal lesions using non-ionizing, safe radiation (light).

Strategies for managing dental caries have increasingly used the concept of risk assessment [4-6]. Caries risk assessment is the determination of the likelihood incidence of caries in the immediate future, and aims to predict the rate of progression of current non-cavitated carious lesions [7]. Dentists can help prevent cavitation of early incipient enamel lesions by correctly identifying key risk factors and demineralization in the early stages [8,9]. The presence of non-cavitated lesions have been shown to have a predictive value [10] for future caries elsewhere. Detecting near-surface incipient lesions allow arresting or reversing the disease progression through topical therapies and improved oral hygiene and diet, as highlighted in CAMBRA caries management strategies [11]. For patients with incipient non-cavitated lesions that have progressed just into the outer surface of dentin, sealant placement or minimal invasive restorations are advantageous to arrest the lesion [12]. Risk assessment is now viewed as the critically important step in the clinical decision process of managing the disease. For teledentistry, caries risk assessment can effectively manage and triage patients for treatment planning, prevention, and establishing follow-up and recall times.

Increasing the accuracy of detecting and assessing early carious lesions with either objective direct measurements or an imaging modality that can allow remote assessment would serve children in critical communities. A potential objective caries detection measurement that could be collected by adjunct personnel may be accomplished using readings from the visible light based Midwest Caries ID™ (Dentsply, York, PA) [13-15]. Conceivably, adjunct personnel may electronically send a table of recorded readings from Midwest Caries ID™ to aid an offsite dentist in treatment planning. Another emerging method for teledentistry includes near infrared imaging modalities, such as Cross-Polarization Optical Coherence Tomography (CP-OCT) [16,17]. CP-OCT can be compared conceptually to ultrasound imaging since both techniques use an incident beam and measure a reflected or backscattered signal. Conventional OCT and the polarized enhanced CP-OCT has been shown to detect early carious lesions through an increase in light scattering [18-20]. CP-OCT is promising because it performs a real time optical image of the microstructure of the tissue, without radiation or surgical intervention [18,19,21]. Multiple OCT images can be produced within seconds, which is advantageous as a clinical detection technique [18]. The rapid acquisition of CP-OCT imaging can allow adjunct dental personnel to use the device for preventive dental screenings. Acquired CP-OCT images can later be interpreted by a dentist; however, there are few comparisons between other less costly optical devices, such as the Midwest Caries ID™ (MID) and simple visual camera images (CAM).

The purpose of this study was to simulate a teledentistry assessment using an *ex vivo* extracted tooth model that could be histologically validated after tooth sectioning. The novelty of this study, compared to previous CP-OCT work, was that a blinded study design was employed, and the primary aim was to compare findings of CP-OCT with less costly options such as the MID and CAM. In addition, this study aimed to determine the examiner reliability of these caries assessment methods to determine their suitability for teledentistry.

## Methods

The Human Subjects Committee from the University of Minnesota (Study # 1002E77235) determined that this study was exempt from review based on the protocol that extracted permanent posterior teeth were collected at local oral surgery offices without patient identifiers. A single examiner sorted through collected teeth and chose an assortment of teeth without evidence of cavitated lesions (ICDAS-II 0–2). A total of 45 samples were initially used. Additional inclusion criteria for teeth in this study were an apparent absence of developmental defects, fluorosis, occlusal restorations and fissure sealants. During the collection process the teeth were stored in 0.1% thymol solution. Before being stored in 0.1% thymol solution the teeth were cleaned to remove all debris. The teeth were then sterilized, using a 5% hypochlorite solution for 10 minutes, and stored back in a 0.1% thymol solution to prevent desiccation of the teeth.

**Figure 1:**
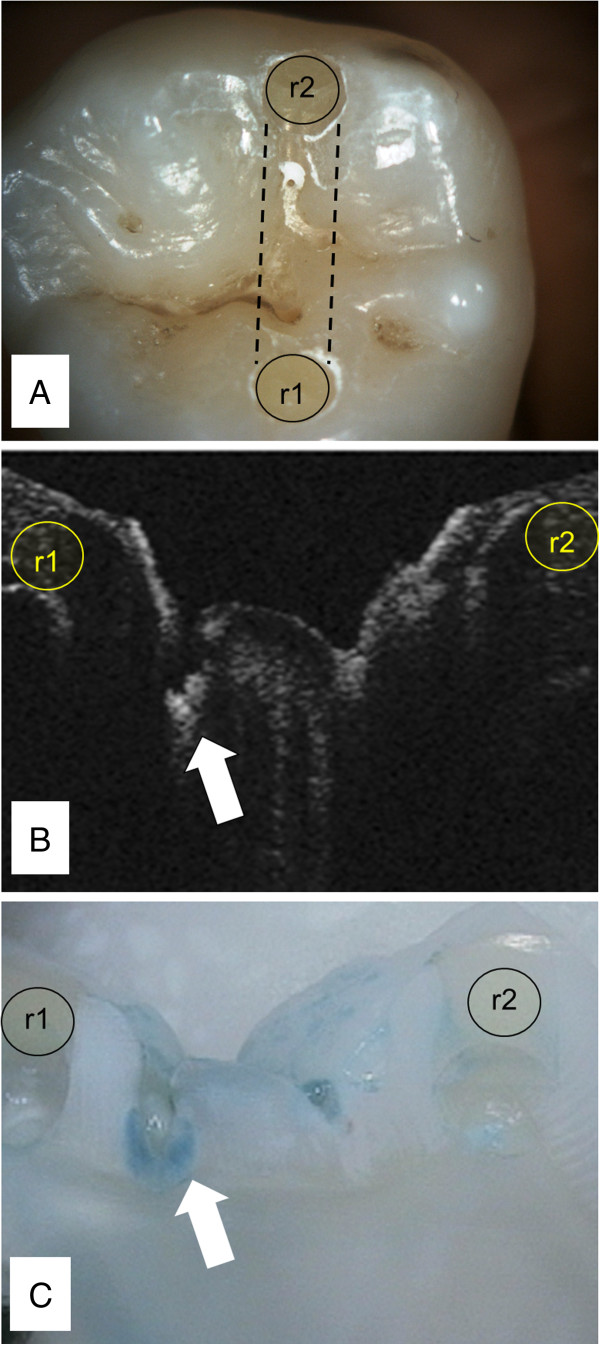
**Each tooth had two composite reference points (r1 and r2). **The test area was the fissure area between the points. **A**) All CAM images were assessed Score ‘2’. All MID Scorings were ‘0’. **B**) CP-OCT images were taken before tooth sectioning. CP-OCT images are presented in grayscale. Black areas have low scattering and white areas have high scattering. All CP-OCT scorings were ‘2’ based on the fissure area near r1 (arrow). **C**) Histological evaluation using caries indicator dye confirmed demineralization extending into enamel and dentin with Score of ‘2’ (arrow).

Teeth were cleaned with pumice slurry to simulate a ‘prophy cup’ cleaning prior to assessment and copiously washed with water. Reference points were placed in each tooth to allow a specific occlusal area to be reproducibly examined by multiple diagnostic methods. Reference points (~0.5 mm diameter × 1.5 mm deep) were small restorations (A5 Accolade SRO^®^, Danville Materials, San Ramon, CA). The line connecting the lingual and buccal reference points was the area of examination for all methods of detection (Figure 1A).

### Study design

**Figure 2:**
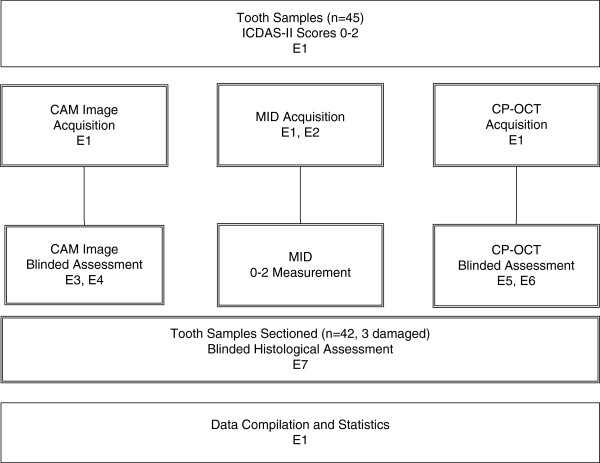
**Ex vivo experimental design. **A total of seven blinded examiners (E) were used for the study. Examiner 1 (E1) initially sorted the tooth samples, collected one of the MID readings and obtained CAM and CP-OCT images, and compiled the results. E1 was blinded to the other examiners (E2-E7) assessments prior to recording the MID readings. E2-E7 were blinded. E5 and E6 did not have access to any visual images of the tooth samples and made assessments solely on the CP-OCT images provided.

This study utilized six blinded examiners (Figure 2) to simulate the work flow of a teledentistry assessment where images or measurements were acquired and later evaluated. All examiners were dentists. The first examiner performed the sorting of the teeth and acquired visual camera images. This first examiner (E1) performed measurements of the Caries ID™ MID prior to any visual camera assessment and CP-OCT imaging to reduce reporting bias of the MID results. Examiner 1 also performed the MID measurement prior to Examiner 2, who also examined the teeth using the MID. Examiner 1 acquired the CP-OCT images. Examiners 2–6 were fully blinded to any other assessment (Figure 1). Examiners (E3, E4) assessed the acquired visual camera images (CAM). Both CP-OCT examiners (E5, E6) were only given the CP-OCT images for assessment. Each examination method was repeated after one to two weeks in order to determine intra- and inter-examination reliability. After caries assessment from Examiners 1–6, teeth were histologically sectioned and assessed by a seventh examiner (E7). E7 was blinded to all the other examiner assessments and images. After histological assessment, E1 was then unblinded to E2-E7 assessments. E1 compiled the assessments for statistical analysis.

In previous studies, ICDAS-II score 1 and 2 teeth have both been shown to have demineralization extending into the dentin in approximately 17-33% of *in vitro* cases [22-24]. In this study, we asked our examiners to assess the occlusal samples (ICDAS-II score 0–2) and determine the presence and depth of demineralization. The visual assessment (CAM) acted as a ‘baseline’ for comparison since there is no established methodology in assessing depth from ICDAS-II scores. This study examines whether MID and CP-OCT improve upon the limitations of trying to deduce depth through visual assessment. Examiners were asked to score the teeth, by each of the three diagnostic methods, in a manner that related directly with possible clinical recommended treatment options (Table 1). This methodology is similar to those employed by past studies examining how lesion assessment translates to treatment decisions by an examiner [25,26] and Aktan et al. study examining the MID to deduce depth in occlusal lesions [15]. For this study, a score 0 indicated that the tooth appeared sound with no evidence of demineralization. This corresponds to a CAMBRA recommendation to address risk factors [11,27,28]. A score ‘1’ indicated that the examiner assess the presence of an enamel-only occlusal caries lesion. The likely treatment option applied clinically would be to determine key biological and behavior risk factors and consider topical therapies (e.g. fluoride based) to promote lesion remineralization [11,27,28]. A score ‘2’ indicated examiner evaluation of occlusal demineralization that progressed into the dentin. The likely treatment option would be to determine risk factors for future caries, deliver appropriate prevention therapy, and seal or restore the lesion with minimal intervention [27,29].

**Table 1:** Threshold scores for gold standard histology and corresponding CAMBRA based recommendations relating to specific lesion depth thresholds for occlusal decay

**Score**	**0**	**1**	**2**
Histology (after sectioning)	Sound, No Evidence of Demineralization	Demineralization Extending into Enamel only	Demineralization Extending into Enamel and outer Dentin
CAMBRA based Recommendations	Assess/Address	Assess/Address Risk Factors	Assess/Address Risk Factors
	Risk Factors	Arrest/Remin Early Lesion with Therapeutics	Treat with Minimal Intervention (e.g. sealant or small restoration)
MID	Green/No Beep	Red/Low Frequency Beep	Red/High Frequency Beep
CAM	Sound Assessment	Demineralization in Enamel Assessment	Demineralization Extending into Dentin Assessment
CP-OCT	Sound Assessment	‘Incipient Subsurface’	‘Extensive Subsurface’
		Demineralization Assessment	Demineralization Assessment

Threshold scores for current and potential teledentistry assessment methods: Midwest CariesID™ (MID), and 6x magnification camera images (CAM), and near infrared based CP-OCT.

### Visual method

Each tooth was photographed twice with a camera/microscope set-up to optimize the lighting and contrast of our *ex vivo* study. The camera (Spot Insight QE Model 4.2^®^, Diagnostic Inc., Sterling Heights, MI) was attached to a SMZ-2 T^®^ microscope (Nikon, Tokyo, Japan) at 6X magnification, which used a fiber optic light source DYNA Light DL-150^®^ (A G Heinze CO, Irvine, CA). This set-up reduced, but did not eliminate, specular (surface) reflection as a confounding variable for our *ex vivo* teledentistry simulation. The first photograph was taken after placing one drop of Hanks Balanced saline solution on the occlusal tooth surface of the moist tooth. This was done in order to mimic natural saliva on a tooth in the oral cavity. The second photograph was taken after prolonged air-drying (approximately 10 seconds). The teeth were then stored back in a 0.1% thymol solution. Examiners (E3, E4) were shown visible images (referred as CAM) of both the moist and dry samples. The area between the reference points were then scored by two examiners (Table 1, Figure 2).

### Midwest Caries ID™

The two examiners (E1, E2) were trained to use the Midwest Caries ID™ (MID) according to the manufacturer’s directions. The MID uses a Light-Emitting Diode to emit a range of red light and then measures the scattering of light due to mineral changes experienced from carious lesions to determine if carious lesions exist [14,15]. Red light normally scatters in sound tissue in a predominantly forward direction with minimal light being backscattered. As the mineral content of the measured area becomes less mineralized, more back scattering is detected, since demineralization causes isotropic (360 degree) scattering [30]. MID detects this increase in backscattering but there are few studies examining how much backscattering intensity is interpreted as caries. The individual measurements with the MID device occurred after calibration of the device. Teeth were kept moist. The tip of the device was inserted vertically on the surface of each tooth and moved around slightly (without pressure) in the pits and fissure area between the reference points. The MID uses a red indicator light to communicate to the examiner the presence of demineralization [15]. In addition the MID produces sound at two frequencies; with the higher frequency designed to detect higher scattering being associated with a larger more extensive amount of decay [15]. The highest reading (Table 1) was recorded for the test area of each sample (Figure 1). Only one measurement was recorded for each tooth.

### CP-OCT imaging

CP-OCT is a non-destructive imaging system that can utilize near-infrared (NIR) light to produce depth resolved images in dental enamel. NIR illumination, especially near 1310 nm, significantly improves the axial imaging depth over wavelengths in the visible range, since dental enamel has been shown to be nearly transparent to NIR light. Using the reference points as a guide, each tooth was imaged with a single tomogram (b-scan), under moist Hank’s Balance saline solution conditions, with a custom cross-polarization swept source OCT (CP-OCT) system with an intraoral probe (IVS-200-CPM, Santec Co. Komaki, Japan). Details of the system and image processing are described elsewhere [31]. The free space axial resolution for the source was experimentally measured to be 11 μm [31]. The probe optics used a low numeric aperture (NA) lens to maximize a Rayleigh range of approximately 4 mm in order to accommodate imaging the complex tooth morphology and image deeper structures like the DEJ. The tradeoff was an 80 μm lateral resolution (1/*e*^2^) that was confirmed using a digital caliper [31]. The focus point for the samples imaged was near the occlusal surface. CP-OCT images were presented to two blinded examiners who had at least 4 months experience assessing CP-OCT images of sound, non-cavitated, and cavitated lesions. Examiners (E5, E6) independently graded the images (Table 1, Figure 2).

### Histology validation and statistical analysis

After Examiner 1–6 performed their blinded assessments, the teeth were sectioned longitudinally (using references points as guides) to approximately 0.5 mm in thickness with a water cooled diamond blade on a Isomet 5000 Linear Precision Saw^®^ (Buehler, Lake Bluff, IL). The sectioned tooth surface was then colored with a glycol based caries indicator dye, To Dye For^®^ (Roydent Dental Products, Johnson City, TN), using a brush and rinsed in tap water for 10 s to remove the dye remnants. This technique is based on a previous study examining early occlusal demineralization and the MID [13]. The histology sections were graded by blinded Examiner 7 (Table 1, Figure 1).

All detection methods were compared by calculating (MedCalc Software, Belgium) the sensitivity, specificity, examiner reliability, and a receiver operator curves (ROC) for each individual diagnostic method; Visual Image Exam (CAM), Midwest Caries ID™ (MID), and CP-OCT. In order to evaluate the diagnostic ability of each examiner, ROC were plotted. A ROC plots ‘sensitivity’ and ‘1-specificity’ values at different threshold values of the examiner or operators receiving the test information. The gold standard disease threshold is kept constant for each point in the plot. The main purpose of an ROC is to test how different threshold values of the diagnostic test affect sensitivity and specificity. For each diagnostic test, DeLong’s method of ROC analysis was used to calculated a P-value and measure the probability that the observed area under the ROC curve was different than a random or ‘coin flip’ diagnostic test (null hypothesis Area = 0.5) [32]. A summary of ROC analysis is described elsewhere [33]. ROC analysis helps determine if an operator systematically over or under evaluates the diagnostic test or image. For intra-examiner reliability, an unweighted kappa statistic was calculated to determine the agreement of each of the examiners between their first and second attempt. For a comprehensive intra- and interexaminer reliability assessment for each detection method, the Intraclass Correlation Coefficient (ICC) was calculated and represented the absolute agreement of all four readings for each diagnostic method.

## Results

Figure 1A shows a sample with the two composite reference points (r1 and r2) that were added to allow the CAM, MID, and CP-OCT analysis to represent similar areas of the tooth sample. In Figure 1A, the CAM image of the pit and fissure region was assessed by E3 and E4 to have demineralization that extended past the DEJ. E3 and E4 graded this pit and fissure region a score ‘2’ at both scoring attempts, which were 1–2 weeks apart. In contrast, E1 and E2 measured a Score ‘0’ from the MID on both attempts. The CP-OCT image (Figure 1A) scoring were ‘2’ by E5 and E6 on both attempts. CP-OCT images (Figure 1B) were taken before tooth sectioning and represented a tomographic image of the select area shown in Figure 1A. The CP-OCT images were all presented to examiners in grayscale. Black areas have low scattering and white areas have higher scattering. Figure 1C illustrates how the reference points allowed for precise sectioning and histological validation of the extent of the demineralization extending past the DEJ of the sample.

Histology assessment determined that 10 samples were sound, 19 samples had demineralization extending into enamel only, and 13 samples had demineralization extending into enamel and outer dentin. From the histological assessment, diagnostic contingency tables were created for each diagnostic test and examiner attempt. The Sensitivity (Sen) and Specificity (Sp) diagnostic values for each examiner attempt were then calculated (Table 2). To clarify notations, ‘MID_E1a’ represents the first attempt of Examiner 1 to assess the samples with MID. MID_E1b represents the second attempt of Examiner 1 performed 1–2 weeks later. Diagnostic values are reported at two disease thresholds defined after histological sectioning. The first threshold examines differentiating any type of demineralization (score 1,2) versus sound enamel (score 0). Sen and Sp values (Table 2, left hand column) were calculated using both score 1 and 2 defining disease. A second threshold was also used to determine the ability to differentiate demineralization extending into enamel and outer dentin (score 2) versus earlier stages (score 0,1). These Sen and Sp values (Table 2, right hand column) were calculated using score 2 defining disease.

**Table 2:** Sensitivity (Sen) and specificity (Sp) diagnostic values for each examiner attempt

	**Disease defined as score 1,2**		**Disease defined as score 2**	
	**Sen**	**Sp**	**Sen**	**Sp**
MID_E1a	50.0	80.0	15.4	82.8
MID_E1b	50.0	80.0	23.1	86.2
MID_E2a	43.8	90.0	15.4	93.1
MID_E2b	43.8	90.0	15.4	89.7
Mean MID	46.9	85.0	17.3	88.0
CAM_E3a	71.9	60.0	53.8	51.7
CAM_E3b	81.3	60.0	53.8	62.1
CAM_E4a	84.4	30.0	46.2	55.2
CAM_E4b	84.4	60.0	38.5	62.1
Mean CAM	80.5	52.5	48.0	57.8
CP-OCT_E5a	75.0	70.0	38.5	80.4
CP-OCT_E5b	81.3	60.0	53.8	72.4
CP-OCT_E6a	84.4	20.0	23.1	75.9
CP-OCT_E6b	93.0	30.0	61.5	62.1
Mean CP-OCT	83.4	45.0	44.2	72.7

To clarify notations, MID_E1a represents the first attempt of Examiner 1 to assess the samples with MID. MID_E1b represents the second attempt of Examiner 1 performed 1–2 weeks later. Diagnostic values are reported at two disease thresholds defined after histological sectioning. Mean values of the Sen and Sp are reported.

The sensitivity and specificity values for both thresholds were used for each diagnostic method to create receiver operator curves (ROC) for each examiner attempt. A summary of the area under the curve (AUC) for each ROC is shown in Table 3. The left column of Table 3 shows the ROC plotted with the gold standard disease threshold at any lesion (score 0 versus 1,2). Several examiners had AUC values that showed the ability to assess demineralization greater than a random test (P < 0.05). The right column of Table 3 shows the ROC plotted with the gold standard disease threshold at score 2. No examiner had an AUC that was significantly different from a random (coin flip) test in assessing demineralization extending into dentin.

**Table 3:** Receiver operator curve analysis

	**Histological 0 versus 1,2**		**Histological 0,1 versus 2**	
	**AUC**	**p-value**	**AUC**	**p-value**
MID_E1a	0.64	0.093	0.61	0.21
MID_E1b	0.67	0.017*	0.63	0.13
MID_E2a	0.68	0.0058*	0.63	0.12
MID_E2b	0.68	0.0048*	0.63	0.14
CAM_E3a	0.66	0.083	0.56	0.46
CAM_E3b	0.68	0.095	0.61	0.20
CAM_E4a	0.62	0.23	0.52	0.83
CAM_E4b	0.77	0.0009*	0.59	0.31
CP-OCT_E5a	0.73	0.0083*	0.58	0.38
CP-OCT_E5b	0.75	0.0026*	0.62	0.21
CP-OCT_E6a	0.59	0.27	0.55	0.58
CP-OCT_E6b	0.71	0.020*	0.62	0.17

AUC is the area under the ROC curve. P-values represent whether the AUC is statistically different from 0.50, which represents a random 50/50 or ‘coin flip’ diagnostic test. Asterisk (*) denotes p < 0.05 significance.

To assess examiner agreement and reliability when assessing sound versus any demineralization (score 0 versus 1,2), unweighted kappa values and Intraclass Correlation Coefficient (ICC) were tabulated (Table 4). Unweighted kappa statistic determined the agreement of each of the examiners between their first and second attempt. The ICC represents the absolute agreement of all four readings for each diagnostic method. MID was found to have the highest kappa and ICC. CP-OCT_E5 had a high kappa value comparable to MID but the overall CP-OCT ICC indicated that there was high variability in assessing the images.

**Table 4:** Examiner agreement and reliability when assessing sound versus any demineralization

	**Score 0 vs. Score1,2**	
	**Kappa (95% CI)**	**ICC (95% CI)**
MID_E1	0.85 (0.68-0.97)	0.83 (0.75-0.90)
MID_E2	0.86 (0.71-1.0)	
CAM_E3	0.56 (0.36-0.75)	0.75 (0.64-0.84)
CAM_E4	0.49 (0.27-0.70)	
CP-OCT_E5	0.72 (0.54-0.89)	0.49 (0.34-0.65)
CP-OCT_E6	0.46 (0.25-0.68)	

Unweighted kappa statistic determined the agreement of each of the examiners between their first and second attempt. The Intraclass Correlation Coefficient (ICC) represents the absolute agreement of all four readings for each diagnostic method. 95% Confidence Intervals are presented.

## Discussion

Optical caries detection has the potential to be incorporated in telehealth medicine for preventive screening; however, this study found that MID and CP-OCT did not have markedly superior diagnostic values from simple CAM assessment. Although MID and CP-OCT were useful in detecting the presence of demineralization, examiners were not able to utilize these devices to adequately assess the depth of the demineralization. The limitations in diagnostic assessment of MID, CAM, and CP-OCT are apparent in the AUC values for each examiner (Table 3). MID and CP-OCT were shown in three out of four examiner assessments, to differentiate a sound pit and fissure region from *any* demineralization. In this study, CAM had only one examiner who could assess the images to yield an AUC value that indicated that the assessment was significant from a random (coin flip) test. The confounding factor of occlusal staining may have reduced the diagnostic utility of CAM. But the difference between the MID, CAM, CP-OCT AUC values were remarkably close. In addition, examiners using MID, CAM, or CP-OCT were not found to be able to properly assess demineralization extending into the dentin (score ‘2’) versus enamel only decay (score ‘1’) and sound enamel. This was shown in the high P-values of the AUC (right column Table 3) curve in comparing the diagnostic values versus a random (coin flip) test.

Applying the results of this study to a clinic teledentistry environment would raise question about the usefulness of either MID and CP-OCT to guide treatment decisions remotely. The concern is that non-cavitated lesions at different stages ideally would be treated by different means. Demineralization extending into the dentin may benefit from a sealant placement to arrest the incipient decay [12] or a minimally invasive preventive resin placement [34]. These options could be over utilized in scenarios with minor enamel demineralization [34] where CAMBRA based recommendations would indicate addressing the overlying risk factors, such a diet and hygiene, and attempting to remineralize the early ‘near surface’ lesion with topical therapeutics [11,35].

The limitations of CP-OCT can be explained when examining Figure 1. Demineralization caused the incident linearly polarized NIR light to be highly scattered and depolarized. The pit and fissure area near the reference point ‘r1’ (Figure 1B, arrow) indicated extensive demineralization for E5 and E6 that was much greater than small surface demineralization; however, the scattering was so extensive in the upper portion of the lesion that the full extent of the demineralization was not measured by CP-OCT. This was likely because the scattering degraded and attenuated the incident light. The CP-OCT NIR light could not penetrate deep enough through the entire lesion. This observation agrees with past studies where demineralization in enamel hides the underlying tooth structure [36,37]. It is important to point out that although the intensity of the incident light and the sensitivity of the CP-OCT detector can be increased in the future, improving these factors may not be able to markedly improve signal penetration, especially at intensity levels that are safe for diagnostic CP-OCT imaging.

Another limitation in CP-OCT imaging was that it was difficult to assess the fissure depth in reference to the DEJ. The DEJ was not apparent in the majority of the images of this study. This was likely caused from the internal reflection angle (critical angle) of light traveling through enamel back to the detector. To clarify, as the returning (back-scattered) signal traveled back through the enamel toward the tooth surface, the signal could be internally reflected when the occlusal topography angles were greater than 37–54 degrees to incident plane (the range is dependent on the water moisture level). In certain occlusal samples, internal reflection could attenuate the backscattered signal from deeper enamel and dentin layers from returning to the CP-OCT for measurement. Another explanation is that the CP-OCT system could not actively focus well below the surface to acquire the deeper DEJ signal; however, the low NA lens on the probe was designed to capture deeper signals.

In assessing *any* demineralization, CP-OCT showed high variability in the image assessment (Table 2). The ICC value (Table 4) showed that the overall agreement of the four CP-OCT readings (E5 and E6) had the highest variability among the three diagnostic methods; however, E5 showed promise as a potential teledentistry clinician examiner with a high kappa value (0.72), reasonable sensitivity values (> 0.75), and marginal specificity values (>0.60). E6’s lower diagnostic values place concern on the reliability of CP-OCT image assessment across multiple operators. This needs to be further explored. Both CP-OCT examiners had lower specificity values compared to the sensitivity values. In general, CP-OCT had lower false negative rate but likely at a cost of higher false positives. Given the high variability in the subjective assessment of CP-OCT images and the lack of molecular specificity for tissue characterization, future caries detection studies should also consider OCT systems that integrate Polarized Raman Spectroscopy (PRS-OCT) [38]. Whereas several fluorescent based caries detection systems indirectly measure caries severity through fluorescence of porphyrins produced by bacteria, PRS examines the depolarization ratio of light targeting the symmetric P-O stretching vibration (959 cm^-1^) of the phosphate ions found in enamel hydroxyapatite crystals [39]. Importantly there are distinct differences between caries and sound enamel depolarization ratios; in contrast, hypocalcified tissue is quite similar to sound enamel [39] and there is potential for PRS-OCT to improve the specificity in assessing early demineralization.

MID showed the highest agreement and reliability among the diagnostic methods (Table 4) in differentiating *any* demineralization versus sound tooth structure. The reliability scores were similar to those studies of Rodrigues et al. and Aktan et al. [13,15]. For our study, examiners who utilized the MID for caries detection tended to have lower sensitivity and higher specificity than the examiners who utilized CAM (Table 2). The Sen, Sp, AUC, and ICC values of this study were nearly equal to those found by Rodrigues et al. when assessing the ability to differentiate sound enamel versus any demineralization (Score 0 versus 1,2) [13]. Aktan et al. reported much lower Sp values for the MID, more variability in Sen, and near equal AUC [15]. One possible explanation is that our study used a similar histological staining for assessing lesion depth as Rodrigues et al. [15], whereas Aktan et al. relied on the raw histological section to assess depth [15]. In assessing deeper demineralization, both our study and Rodrigues et al. [15] study found nearly identical specificity values for MID (90.0 versus 88.0), which indicates a low false positive rate [13]. However, the Rodrigues et al. study found that the MID yielded a much higher sensitivity value than our study. A possible explanation for this difference in study results was that the teeth selected to be used in this paper were non-cavitated (ICDAS-II Score 0–2), and the dentin involvement in our study may have been much less severe than Rodrigues et al. study [13]. The MID has predefined threshold vales. This study suggests that demineralization with initial dentin involvement may be difficult to differentiate versus near surface enamel lesions. The Rodrigues et al. study may be interpreted to suggest that if the dentin demineralization is severe enough, this decay can be differentiated from enamel- only demineralization with higher sensitivity. However, this study indicated that assessing the severity in depth of non-cavitated lesions remains challenging for examiners using the MID, CAM, and CP-OCT.

## Conclusion

Although MID and CP-OCT were useful in detecting the presence of demineralization, examiners were not able to utilize these devices to adequately assess the depth of the demineralization. This study found that MID and CP-OCT did not have markedly superior diagnostic values from simple CAM assessment for use in teledentistry.

## Competing interests

The Division of Pediatric Dentistry at the University of Minnesota, School of Dentistry received an unrestricted donation of the Caries ID™ from Dentsply and composite material from Danville Materials. The authors themselves declare that they have no competing financial interests. The authors have not consulted with either company regarding this study.

## Authors’ contributions

Both authors made substantial contributions to the study design, analysis of the data, co-wrote and approved the final manuscript.

## Pre-publication history

The pre-publication history for this paper can be accessed here:

http://www.biomedcentral.com/1472-6831/13/16/prepub
